# Non-Dairy Fermented Beverages Produced with Functional Lactic Acid Bacteria

**DOI:** 10.3390/microorganisms10122314

**Published:** 2022-11-23

**Authors:** Medana Zamfir, Iulia-Roxana Angelescu, Catalina Voaides, Calina-Petruta Cornea, Oana Boiu-Sicuia, Silvia-Simona Grosu-Tudor

**Affiliations:** 1 Institute of Biology Bucharest of the Romanian Academy, Splaiul Independentei No. 296, 060031 Bucharest, Romania; 2 Faculty of Biotechnologies, UASVM-Bucharest, 59 Mărăşti Boulevard, 011464 Bucharest, Romania

**Keywords:** functional beverages, lactic acid bacteria, polyphenols, antioxidant activity, non-dairy probiotic products

## Abstract

At present, there is an increasing interest in beverages of non-dairy origin, as alternatives to those based on milk, but having similar health-promoting properties. Fermentation with specific bacteria or consortia may enhance the functionality of these products. In our study, selected lactic acid bacteria, that have been previously shown to possess functional properties (antimicrobial activity, probiotic potential), were used for the fermentation of wheat bran combined with root vegetables. Strains were investigated for their safety, while the obtained beverages were characterized in terms of microbial content, physical, chemical, nutritional, and functional properties. None of the strains harbors virulence genes, but all of them possess genes for survival at low pH, starch metabolism, and vitamin biosynthesis. Three strains (*Lactiplantibacillus plantarum* BR9, *L. plantarum* P35, and *Lactobacillus acidophilus* IBB801) and two substrates (5% wheat bran with 10% red beetroot/carrots) were selected based on a preliminary assessment of the beverage’s sensory acceptability. These strains showed good growth and stability over time in the stored beverages. No enterobacteria were detected at the end of fermentations, while the final pH was, in most cases, below 3.5. Free phenolics, flavonoids, and DPPH scavenging effect increased during fermentation in all drinks, reaching 24h values that were much higher than in the unfermented substrates. Most of the obtained drinks were able to prevent the growth of certain pathogens, including *Listeria monocytogenes* ATCC 19111, *Salmonella enterica* ATCC 14028, *Staphylococcus aureus* ATCC 25923, and *Escherichia coli* ATCC 25922. The obtained beverages would combine the nutritiveness of the raw ingredients with the beneficial effect of fermentation (increasing shelf life, health-promoting effect, pleasant flavor, etc.). They would also fill a gap in the non-dairy probiotics sector, which is constantly increasing due to the increasing number of vegan people or people that cannot consume dairy products.

## 1. Introduction

Fermentation, in particular, lactic acid bacteria (LAB) fermentation is known as one of the oldest preservation methods, applied to various substrates, including milk, meat, fish, vegetables, and cereals. Besides preservation, LAB also contributes to the sensory, nutritional, and functional attributes of the final products [1]. Consumption of fermented foods and beverages implies health-promoting benefits [2,3], which include enhanced digestibility, antipathogenic [4,5,6], antihypertensive [5], antioxidant [7], and immune-stimulating properties [8]. Homemade products, obtained by spontaneous fermentation, may differ in aroma, flavor, texture, and quality due to other environmental factors affecting the process [9]. Therefore, there is an increased concern nowadays to enhance the functionality of these products by standardizing the process and using specific bacteria or consortia with proven benefits [1].

The concept of functional foods was born in Japan in the 1980s. They were developed specifically to promote health or reduce the risk of disease and are considered as those foods, which are intended to be consumed as part of the normal diet (not pills or capsules) and exert their effect when used in amounts that can normally be expected to be consumed in the diet [10,11]. Principal substances that impart functionality to a food/beverage include vitamins, minerals, fiber, omega-3 fatty acids, flavonoids, and probiotic bacterial strains [12]. Attention concerning this category of foods has grown once people became aware of the relationship between diet and health [13,14]. On the other hand, consumers are more and more interested in organic foods and they tend to avoid foods with artificial ingredients [15].

Compared with solid foods, beverages offer the advantages of greater possibility of incorporating bioactive compounds and easy delivery and storage of the final products [16]. Commercially, a wide variety of functional beverages exist, including dairy-based beverages, vegetable and fruit-based beverages, sports drinks, energy drinks, tea and tea-based beverages, and whey and soy proteins-based beverages [11]. Dairy beverages (yogurt drinks, fresh or fermented milk) are considered an excellent way of delivering probiotics. However, there is an increasing interest in beverages of non-dairy origin, as alternatives to those based on milk, but having similar health-promoting properties. These products are requested both by vegans and by lactose-intolerant people or those with a high cholesterol level [17]. In this context, various functional non-dairy beverages that contain probiotics have been launched, including fruit, cereals, soybeans, and vegetable-based beverages [18,19].

Fermented foods and drinks of non-dairy origin represent an important part of the daily food in Romania. Among these, fermented vegetables or cereals are in the top positions. Wheat bran, for instance, is widely used in Romania for the production of *borș*, an acidic liquid used in Romanian cuisine for the preparation of a traditional soup known as *borş* or *ciorbă*, but also consumed as a refreshing drink [20]. Wheat bran is the major by-product of wheat processing, but it has high utilization values because of its nutritional contents, such as enzymes, proteins, carbohydrates, vitamins, and minerals [21], with various health benefits, including: enhanced immunity, improved digestion, and metabolism, or antioxidant activity that leads to protection of normal cells from oxidative damage [22]. Moreover, it has been shown that diets rich in cereals play a crucial role in the prevention of chronic diseases such as cardiovascular disease and certain types of cancer [23].

On the other hand, fruit and vegetable juices are also known for their high nutritiveness and health benefits. Red beetroot, for instance, is known for its antihypertensive, hypoglycemic, anti-inflammatory, hepatoprotective, and antioxidant activity [24,25,26,27]. Recently, it has been shown that the antioxidant activity of many plant-based foods was enhanced by microbial fermentation [28]. Moreover, many vegetables, including red beetroot and carrots are rich in pigments with coloring potential but are seasonal and unstable. Fermentation with selected LAB strains may be used to increase their durability and enhance the pigment contents [29,30].

In this context, the aim of our study was to use selected functional LAB strains for the fermentation of substrates containing both wheat bran and root vegetables (red beetroot or carrots) and to characterize the obtained beverages in terms of microbial content, physical, chemical, nutritional, and functional properties.

## 2. Materials and Methods

### 2.1. Bacterial Strains and Growth Conditions

Bacterial strains used as starters in this study have been previously isolated from various (fermented) foods/drinks, including both dairy and non-dairy products (Table 1). These strains were selected based on their functional properties, such as antibacterial/antifungal activity, production of bacteriocins/surfactants/exopolysaccharides (EPS), or probiotic potential, as mentioned in Table 1. All strains were maintained at −80 °C in MRS broth [31], in the presence of 25% (*v*/*v*) of glycerol for cryoprotection, as part of the Culture Collection of the Department of Microbiology, Institute of Biology Bucharest, Romania. Before use, strains were propagated twice, for 24 h, in MRS broth, at 28 °C (the two *Leuconostoc* strains) or 37 °C (all *lactobacilli*). 

### 2.2. Safety Evaluation of the Bacterial Strains Used as Inoculum

#### Detection of Virulence Genes

LAB strains were screened for the presence of virulence genes *ace* (adhesion collagen protein), *agg* (aggregation), and *asa* (aggregation) by PCR, following the method/primers of Pieniz et al. [37], as mentioned in Table 2. Genomic DNA was extracted from overnight LAB cultures, using a Pure Link Genomic DNA kit (Invitrogen, Carlsbad, CA, USA) according to the manufacturer’s guidelines. The PCR was performed by adding 50 ng/μL of DNA extract to a final volume of 25 µL reaction mixture containing 1× GoTaq Flexi buffer, 2.5 mM MgCl_2_, dNTP Mix (containing 0.2 mM each dNTP), 0.5 µM forward and reverse primer (Table 2), and 1.25 U GoTaq G2 Hot Start Polymerase (Invitrogen, Waltham, MA, USA). The PCR conditions for virulence genes, *ace*, *asa*, and *agg*, were as follows: initial denaturation at 92 °C for 2 min followed by 35 cycles of denaturation at 92 °C for 30 s, primers annealing at optimum temperature for each set for 1 min (Table 2), 72 °C extension for 1 min and final extension at 72 °C for 5 min. The PCR products were analyzed on 1.5% agarose gels supplemented with SYBR Safe DNA Gel Stain (Invitrogen).

### 2.3. Genetic Screening of LAB Functional Properties

The bacterial strains used as inoculum were screened by PCR multiplex reactions for the presence in their genome of some functional genes involved in stress resistance, production of vitamins, and starch metabolism, respectively. The function of each investigated gene and the primers used for the screening are listed in Table 2. KAPA2G Fast Multiplex PCR kit (KAPA Biosystems, Wilmington, MA, USA) was used and each mixture (25 µL) contained 2× multiplex mix, 10 µM of each primer, and 100 ng DNA template. The PCR conditions were as follows: initial denaturation at 95 °C for 3 min followed by 30 cycles of denaturation at 95 °C for 15 s, primers annealing at a temperature depending on the primer (Table 2) for 30 s, elongation at 72 °C for 90 s, and a final elongation at 72 °C for 10 min. PCR products were separated on a 2% agarose gel, supplemented with ethidium bromide, and UV-analyzed for the presence of a unique amplicon. 

### 2.4. Fermentation of Wheat Bran and Root Vegetables with Selected LAB Strains

Various combinations of wheat bran (WB) with either red beetroot (BR), carrots (C), or both, were used as fermentation substrates (Table 3). Both WB and vegetables were purchased from local markets. Fresh BR and C were blended, added to the WB, and suspended in hot water. After cooling down to about 40 °C, each combination was inoculated (2%) with one of the LAB strains listed in Table 1. For this, bacterial cells were recovered from overnight MRS cultures by centrifugation (10,000× *g*, 4 °C, 10 min), washed twice with saline water, and suspended in saline water at a concentration of about log 8–9 CFU/mL before being inoculated. Mixtures were incubated for 24 h at the optimal growth temperature of each bacterial strain. 

Based on a preliminary sensory analysis (data not shown) of the fermented products, two substrate combinations (numbers 3 and 4 from Table 3), inoculated with *L. plantarum* BR9, *L. plantarum* P35, and *L. acidophilus* IBB801 (2% inoculum prepared as above), or various mixtures of them (1% inoculum of each strain), were selected for further studies. During fermentation, 50 mL samples were taken from each batch at 0, 3, 6, 9, 12, and 24 h for analyses.

### 2.5. Microbiological Analyses

LAB viability was evaluated by counting the CFU numbers on MRS agar plates (1.5% agar), in each sample collected during fermentation. The viability of the starter strains was further determined 1 week after the end of fermentation, during storage at 4 °C.

Undiluted samples were also plated on violet-red-bile-glucose (VRBG) agar medium (Merck KGaA, Darmstadt, Germany) supplemented with 0.1 g/L of cycloheximide (incubated at 37 °C), and yeast extract-peptone-glucose (YPG) agar medium (Merck) supplemented with 0.1 g/L of chloramphenicol (incubated at 28 °C), for the enumeration of enterobacteria and yeasts, respectively. 

### 2.6. Physical, Chemical, and Nutritional Characteristics

pH measurements. Acidification during the fermentation was analyzed by measuring the pH with an InoLab 720 pH meter (WTW, Weilheim, Germany).

Lactic acid production. A Jasco HPLC System (Jasco Europe, Cremella, Italy), equipped with a PRPx300 (Hamilton, Switzerland) column, maintained at 60 °C, and coupled with a photodiode array (PDA) detector, was used for lactic acid quantification. Elution was performed with 2.5 mM H_2_SO_4_, at a flow rate of 0.5 mL/min. 

Free phenolics. The free phenolic content was measured using the Folin–Ciocalteu method [39] and the results were expressed as µg of gallic acid equivalents per ml of sample (µg GAE/mL).

Flavonoids content. The content of total flavonoids was assessed based on the method described by Dewanto et al. [40]. Briefly, 1 mL of each sample was mixed with 4 mL of deionized water and 0.3 mL of 5% NaNO_2_. After 5 min at room temperature, 0.3 mL of 10% AlCl_3_ was added to the mixture and kept for another 6 min. Finally, 2 mL of 4% NaOH and 2.4 mL of deionized water were added and the absorption was read after 15 min at 510 nm against water. Rutin was used as standard, and results were expressed as µg of rutin equivalents per ml of sample (µg RE/mL).

DPPH free radical scavenging activity. The free radical scavenging activity was determined using DPPH (2,2-diphenyl-1-picrylhydrazyl radical), based on the method of Moon and Terao [41]. Briefly, 0.1 mL of sample was added to 2.25 mL methanol and vortexed. DPPH (1.27 mM) was then added (0.15 mL), vortexed again, and incubated for 30 min in the dark, at room temperature. The absorbance was finally measured at 517 nm, using a control without sample. The scavenging effect (Se) was calculated using the formula [42]:

(1) $$Se\;\left(\%\right)=1-\frac{Abs\;sample}{Abs\;control}\times 100$$

where *Abs* means absorbance of the sample/control.

All determination were done in triplicate and the results are given as mean value ± standard deviation (SD).

### 2.7. Functional Properties

Antibacterial activity. The antagonistic activity of the samples collected during fermentation was assayed by the agar spot method [43] against *L. delbrueckii* subsp. *bulgaricus* LMG6901^T^ as indicator strain. Samples were centrifuged to remove cells, and the supernatant was serially diluted up to 1/8. From each dilution, 10 µL were spotted onto a fresh indicator lawn (0.7% agar MRS with 100 µL of the sensitive strain). The activity was defined as the reciprocal of the highest dilution for which a clear inhibition zone could be observed, and was expressed in activity units (AU) per milliliter of sample. 

Prevention of pathogens growth. Aliquots of the fermented drinks were filter sterilized and inoculated with one of the following pathogens: *Listeria monocytogenes* ATCC 19111, *Salmonella enterica* ATCC 14028, *Staphylococcus aureus* ATCC 25923, *Escherichia coli* ATCC 25922, and *Bacillus cereus* CBAB, in two different concentrations (2%, and 10%, respectively). OD_600nm_ was measured hourly, for 24h, in a plate reader (Spectra Max, Molecular Devices, San Jose, CA, USA). Filter-sterilized non-fermented substrates inoculated with the pathogens served as controls.

### 2.8. Statistical Analysis

Experimental data were recorded as mean ± standard deviation of triplicates, generated by GraphPad Outlier calculator. Outliers were calculated using a significance level (alpha) set at 0.05. GraphPad Prism (GraphPad Software LLC, San Diego, CA, USA) was used for the statistical analysis of the results (One-way ANOVA with a post hoc test: Dunnett’s multiple comparisons test), also with a significance level (alpha) set at 0.05. 

## 3. Results

### 3.1. Safety Evaluation of the Bacterial Strains Used as Inoculum

PCR reactions with three primer sets used for the detection of virulence genes did not result in specific amplification for any of the genomic DNA extracted from the seven tested strains (Figure S1). 

### 3.2. Genetic Screening of LAB Functional Properties

As seen in Table 4, most LAB strains selected for this study harbor genes involved in survival at low pH (*LBA1272*), in starch metabolism (*agl, α-amy*, and for some strains *malL*), and in vitamin production (*folP* and *ribA*). None of the strains harbors *dltD* or *folK* genes.

### 3.3. Fermentation of Wheat Bran and Root Vegetables with Selected LAB Strains

#### 3.3.1. Microbiological Analysis

LAB counts were variable at the start of the fermentation, ranging from about 6 to about 8 log CFU/mL, the highest being for *L. plantarum* P35 (Table 5). The counts increased fast during the first 6h for all cultures, and reached, after 24h, about 8 log CFU/mL in the case of *L. acidophilus* IBB801, and about 9 log CFU/mL for the other strains, regardless the substrate used for fermentation. A slight difference could be detected in the case of *L. plantarum* P35, both as single culture, and in combination with *L. acidophilus* IBB801, for which the growth was faster when carrots were used instead of red beetroot. 

The presence of yeasts was not detected in any sample (results not shown), but low numbers of enterobacteria (up to about 3 log CFU/mL) were counted in the single cultures of *L. plantarum* BR9 and *L. acidophilus* IBB801, at certain times, in the middle of the fermentation. No enterobacteria were present at the end of the fermentation (24 h) when the liquid was removed and stored at 4 °C. 

After one week of storage, the CFU counts decreased with a maximum of 1.4 log CFU/mL in the liquids obtained from the carrots fermented with the single cultures of *L. plantarum* BR9, but there were drinks in which CFU counts were not affected (Table 5). 

#### 3.3.2. Physical, Chemical, and Nutritional Characteristics

##### PH Value

During the fermentations, the pH drop was fast, starting within the first 3 h, as the bacterial growth occurred. At 24 h, the pH reached values of about 3.0 in most cases (Table 5). The highest pH values measured at 24 h (3.4–3.6) were for the single culture of *L. acidophilus* IBB801, in both substrates.

##### Lactic Acid Production

Lactic acid could not be detected in the first 3 h of fermentation in any of the collected liquids. At 6 h, small amounts of lactic acid were present in almost all samples. The concentration increased afterward, directly related to bacterial growth (Table 5). *L. plantarum* P35 and *L. acidophilus* IBB801, both in single cultures and in their co-culture, produced more lactic acid in the substrate based on carrots, where the growth was also faster. However, *L acidophilus* produced the lowest amount of lactic acid (about 8 mg/mL at 24 h), compared to the other strains (between about 11 and 15 mg/mL at 24 h).

##### Free Phenolics

**Figure 1:**
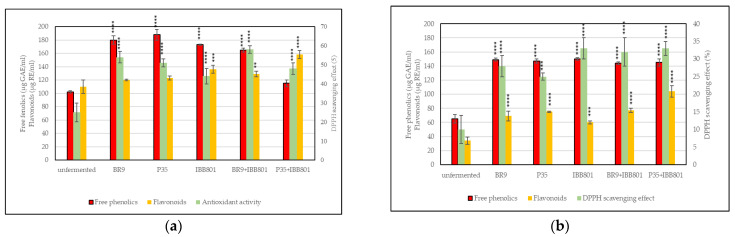
Free phenolics, flavonoids, and DPPH scavenging effect of the fermented beverages: (**a**) WB + BR; (**b**) WB + C. Differences between fermented and unfermented samples were statistically analyzed (One-way ANOVA with post hoc test: Dunnett’s multiple comparisons test) and *p* value summary was marked with asterisks on the graph; no asterisk means no significant difference, while **, ***, and **** mean *p* < 0.01, *p* < 0.001, and *p* < 0.0001, respectively.

Total free phenolics were higher when red beetroot was used as a substrate, and increased during fermentation from 112–128 µg GAE/mL, immediately after inoculation, to about 158–188 µg GAE/mL, at 24 h (about 50% increase) (Table 5). When carrots were used in combination with wheat bran, total free phenolics were lower during the whole fermentation process, but the increase in the end samples was over 75%, and even over 100% in the case of *L. acidophilus* IBB801 compared to the initial values. Moreover, all end values were higher than the ones of the unfermented substrates, regardless of the vegetable type (Figure 1).

##### Flavonoids Content

The flavonoid content was higher in the fermented substrates than in the unfermented samples, but the amounts depended on the vegetable and on the strain used as inoculum (Figure 1). In the beetroot fermentations, the initial values ranged from about 83 µg RE/mL (for *L. acidophilus* IBB801) to about 116 µg RE/mL (*L. plantarum* P35) (Table 5). However, the increase of the flavonoid content over time was the highest in *L. acidophilus* IBB801 final samples (about 64%) and the lowest in *L. plantarum* P35 final samples (about 6%). In the carrot fermentations, even if lower concentrations were measured, the increase over time was much higher, the lowest being 45% for the co-culture BR9+IBB801, and the highest for P35, in single culture (114%), and in co-culture with IBB801 (148%).

##### Antioxidant Activity

The antioxidant activity determined in the red beetroot fermentations as a DPPH scavenging effect, increased, in general, in the first 3h from inoculation, and remained afterward approximately constant till the end (Table 5). Compared with the unfermented samples, the antioxidant activity is about double (*p* < 0.0001) in all drinks obtained with this substrate (Figure 1a). When carrots were used instead of red beetroot, the antioxidant activity did not register significant changes during fermentation, and the values were, in general, lower (maximum 33%) than in beetroot (maximum 58%). However, the final values, at 24 h (between 25–33%), were significantly (*p* < 0.0001) higher than for the unfermented samples (10%) (Figure 1b).

#### 3.3.3. Functional Properties

##### Antibacterial Activity 

Except for the two variants fermented with *L. plantarum* BR9, all drinks showed antibacterial activity when tested against *L. delbrueckii* subsp. *bulgaricus* LMG6901^T^ as indicator strain (Table 5). In general, the activity was higher in WB + C drinks and reached a maximum of 200 AU/mL. When *L. plantarum* P35 was used in single culture or in co-culture, the activity was detected much earlier (after 3–6 h of fermentation) compared with the other strains (after 9–12 h).

##### Prevention of Pathogens Growth

**Figure 2:**
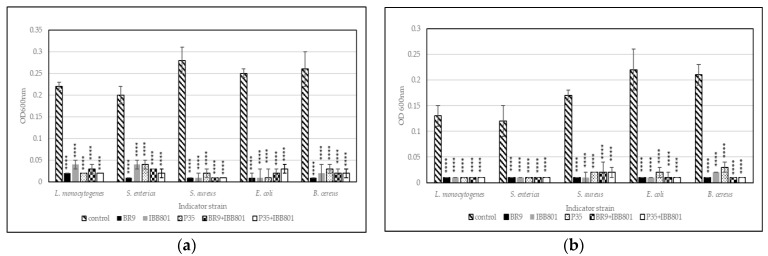
Growth (recorded as maximum OD_600nm_) of pathogens inoculated (2%) in sterilized supernatants of the fermented beverages obtained with: (**a**) WB + BR; (**b**) WB + C. Differences between fermented and control (unfermented) samples were statistically analyzed (One-way ANOVA with post hoc test: Dunnett’s multiple comparisons test) for each pathogen and *p* value summary was marked with asterisks on the graph. **** means *p* < 0.0001.

**Figure 3:**
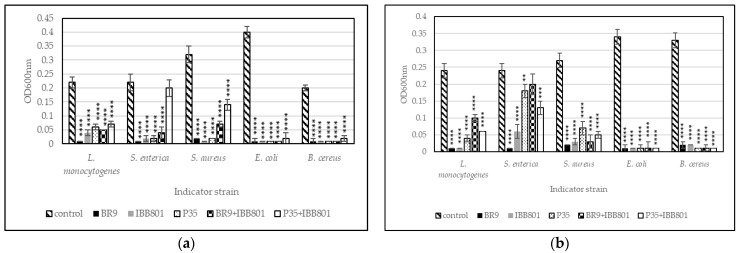
Growth (recorded as maximum OD_600nm_) of pathogens inoculated (10%) in sterilized supernatants of the fermented beverages obtained with: (**a**) WB + BR; (**b**) WB + C. Differences between fermented and control (unfermented) samples were statistically analyzed (One-way ANOVA with post hoc test: Dunnett’s multiple comparisons test) and *p* value summary was marked with asterisks on the graph; no asterisk means no significant difference, while **, ***, and **** mean *p* < 0.01, *p* < 0.001, and *p* < 0.0001, respectively.

Pathogens used in this experiment were able to grow in both substrates used for fermentation (WB+C, and WB+BR, respectively), reaching after 24 h, in general, OD_600nm_ between 0.2 and 0.4, depending on the strain, the concentration of inoculum, and the substrate (Figure 2 and Figure 3). Lower OD_600nm_ values were measured for *L. monocytogenes*, *S. enterica*, and *S. aureus* in WB + C medium, when inoculated in concentrations of 2%.

Figure 2 shows the comparative growth of the five pathogens in control media (WB+BR, and WB+C, respectively) and in sterilized supernatant of the fermented drinks when 2% of the pathogen was used as inoculum. All 10 variants of fermented drinks succeeded at preventing the growth of the pathogens at this concentration, the OD_600nm_ of the culture after 24h being below 0.05.

**Figure 4:**
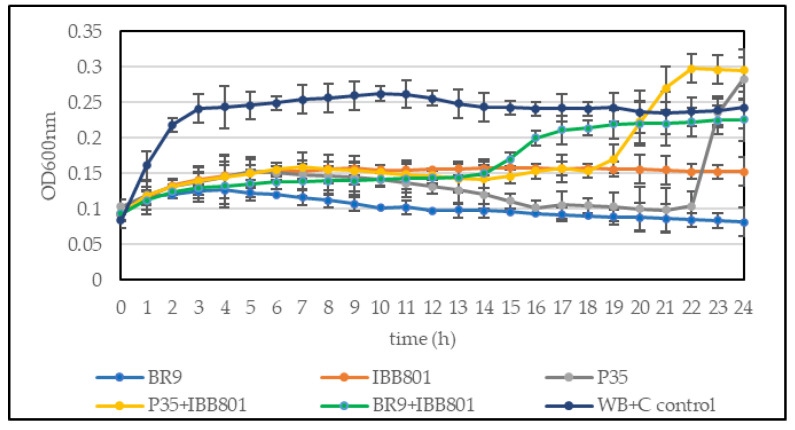
Growth curves of *S. enterica* ATCC 14028 inoculated (10%) in sterilized supernatant of fermented beverages obtained with WB + C. Control: unfermented WB + C.

When a higher concentration of inoculum was used (10%), *E. coli* and *B. cereus* growth was completely inhibited in all drinks (Figure 3). The other pathogens, but especially *S. enterica*, were able to grow in some drinks (such as those obtained with *L. plantarum* P35 and with the co-cultures), up to OD_600nm_ of 0.1–0.2. The values were higher, in general, in the WB + C variants. However, the growth of the pathogens started much later (after 16–20 h) in these drinks as compared with the controls (Figure 4).

## 4. Discussion

Consumption of foods rich in dietary fiber has many health benefits [44]. However, many products, including wastes from cereal processing (i.e., wheat or rice bran), that contain fiber, along with protein, starch, and other beneficial ingredients, are not being fully utilized [45]. One reason is that such products cause lower overall acceptability, darker color, and poor consistency and texture [46]. Fermentation with selected LAB strains may be used to increase the bioavailability of nutrients and to improve sensory properties [47]. 

In general, LAB can be safely included in products intended for human use due to their GRAS (generally recognized as safe) status. In particular, the strains used in our study do not harbor virulence genes and they are sensitive to many common antibiotics, showing their safety for consumption. These LAB strains have been selected based on some particular properties, including antimicrobial activity and probiotic potential, and have been used to obtain fermented beverages using wheat bran as substrate. Red beetroot and carrots were added to this substrate, in order to accelerate bacterial growth and to improve the sensory and nutritional properties of the final products. Indeed, all seven LAB strains were able to grow in all tested combinations of cereals/vegetables, resulting in products characterized, in general, by pleasant flavor and color. Regarding the overall acceptability, the volunteers gave very good scores (over 8) to many of these beverages. Based on their reports, the best two substrates have been selected for further fermentations: 5% WB + 10% vegetable (BR or C). Products obtained with *L. plantarum* BR9 and *L. plantarum* P35 received the best scores on these substrates, due to their strong acidic taste, very similar to the Romanian traditional *borș*. Such fermented products can be succesfully used for the preparation of sour soups. Besides their specific flavor, beverages obtained from WB and BR also have a pleasant color, very well appreciated by most of the volunteers. It has been shown that thermal treatment of BR juice may lead to a change in color to brown [48]. However, fermentation resulted in nice red color, close to that of natural juice, and this may be due to the low pH, of about 4.0, which offers the greatest stability of the red pigment in BR [48]. Fermented drinks obtained with *L. acidophilus* IBB801 were also preferred by many tasters due to the delicate acidic taste, which can be desired in beverages intended to be consumed as refreshing drinks. This strain was used, therefore, in the controlled fermentations, both as single strain and in co-culture with one of the two *L. plantarum*. 

The good bacterial growth in all variants of cereal-based substrates may be explained by the fact that most of the tested LAB strains harbor in their genome genes involved in starch metabolism. Several LAB species, such as *L. amylolyticus, L. amylotrophicus,* and *L. amylovorus*, but also strains of *L. plantarum*, isolated from fermented cassava, maize, sorghum, rice, and beer malt, have been shown to degrade starch in the presence of easier fermentable carbohydrates [49]. 

In the followed-up fermentations, the three selected strains caused a significant decrease of the pH, down to 3.1–3.6, depending on the strain and on the substrate, with a concomitant increase in the viable cell numbers and in the lactic acid production, as it has been previously reported for LAB grown in various plant-based substrates [50]. On the other hand, due to the low pH values, no Enterobacteria were found in the end-products, proving their good fermentation quality. Similar tendencies were reported by Krungleviciute et al. [51] who found that fermentation of barley and wheat bran with LAB reduced the Enterobacteria and total aerobic bacteria count in the substrate. This result can be explained by the drop in the pH value below 3.8, which inhibits most food spoilage bacteria [52]. All the fermented beverages could be stored at 4 °C in well-sealed recipients for a couple of weeks without being spoiled or losing their organoleptic properties, as observed before with laboratory-made *bors* [20]. Moreover, it seems that yeasts were also inhibited by the low pH value since they were not found in any fermented product.

The nutritional characteristics and functional properties of the fermented beverages were also investigated. In general, beneficial health effects of diets rich in cereals have been ascribed to dietary fiber or to some of the components associated with the fiber, including phenolic acids [53]. Together with fibers, polyphenols are important for antioxidant and anti-inflammatory properties. 

Fermentation had an enhancing effect on the total phenolics, flavonoids, and antioxidant activity, in variable degrees, depending on the bacterial strain/combination used as inoculum. The same effect was reported by other studies, using various cereal substrates [54], and it may be explained by activation at low pH of endogenous enzymes of the cereals, causing a structural breakdown of plant cell walls, and a release of bioactive compounds [55,56]. 

Antioxidants (such as vitamin C, phenolic compounds, or glutathione) are able to transform reactive oxygen forms of the oxidoreduction reaction into more stable and non-reactive forms, preventing oxidative cell damage and degenerative diseases [57]. On the other hand, antioxidant enzymes found in plants, including superoxide dismutases, catalases, or glutathione peroxidases, contribute to the detoxification process by converting the reactive oxygen species to hydrogen peroxide, further degraded by peroxidases or catalases to water [58]. It has been also reported that LAB strains might have antioxidant activity themselves, due to the production of antioxidant compounds, such as exo-polysaccharides, peptides, glutathione, benzoic acid, and benzaldehyde, but also antioxidant enzymes, such as superoxide dismutase [57]. This can be the case with our strains since the antioxidant activity determined immediately after inoculation was higher than in the uninoculated samples.

Moreover, total phenolics, but also flavonoids and antioxidant activities, were higher in the red beetroot fermentations than in carrot fermentations. In general, a direct correlation existed between the phenolic content and the scavenging of DPPH radical, as shown by other authors, as well [59]. Therefore, our study reveals the potential of wheat bran and root vegetables, such as red beetroot and carrots, as value-added ingredients for fermented functional foods/beverages. They may be exploited as a potential source of antioxidants in these fermented products and may replace synthetic antioxidants in food formulations [53].

On the other hand, fermented beverages, especially the ones based on red beetroot, can be good matrices for delivering probiotic strains, since they harbor, even after one week of storage at 4 °C, all tested strains, including the potential probiotic *L. plantarum* BR9, at viabilities much higher than log 6 CFU/mL, which is the minimum number for optimal therapeutic effects [60].

Last, but not least, most of the fermented products showed inhibitory activity against several bacterial strains used as indicators. They were able to prevent the growth of pathogens/spoilage bacteria when these were intentionally added (in concentrations up to 10%) to the cell-free supernatants. It is known that LAB are antagonistic toward various bacteria that may be present in raw materials and may cause unpleasant changes in the organoleptic properties [48], and therefore, they may find application in the food industry. This might be also the case for the strains used in this study, especially for *L. plantarum* BR9 and *L. acidophilus* IBB801, which inhibited the growth of most indicator bacteria, even when these were inoculated in a high concentration. Such strains may be used to reduce the amounts of preservatives added to foods, being a much healthier, non-toxic, and non-allergenic alternative to these chemical compounds.

## 5. Conclusions

The strains selected in this study may find application as starters for controlled cereal/vegetable-based fermentations. The obtained beverages would combine the nutritiveness of the raw ingredients with the beneficial effect of fermentation (increasing shelf life, health-promoting effect, pleasant flavor, etc.). They would also fill a gap in the non-dairy probiotics sector, which is constantly increasing due to the increasing number of vegan people or people that cannot consume dairy products.

Products obtained with *L. plantarum* BR9 and *L. plantarum* P35, with a strong acidic taste, are suitable, for instance, as ingredients for sour soups, while the less acidic products obtained with *L. acidophilus* IBB801 may be used as refreshing drinks. All three strains are safe to be used in such products, and their fermentation increases the total phenolic content and antioxidant activity, but also lowers the pH, preventing the growth of undesired bacteria or fungi.

## Figures and Tables

**Table 1:** Bacterial strains used in this study as inoculum for fermentation.

Strain	Isolation Source	Properties	References
*Lactobacillus acidophilus* IBB801	yogurt	antibacterial activity, bacteriocin production	[32]
*Lactiplantibacillus plantarum* BR9	*braga*	antibacterial activity, probiotic potential, antifungal activity ^1^	[33]
*Lactiplantibacillus plantarum* CR1	water kefir	antibacterial activity, probiotic potential, antifungal activity ^1^	[33]
*Leuconostoc mesenteroides* 21.2	milk	exopolysaccharide production	[34]
*Leuconostoc citreum* 52	fermented vegetables	exopolysaccharide production	[35]
*Lactiplantibacillus plantarum* P35	*bors*	antifungal activity, surfactant production, antibacterial activity ^1^	[36]
*Lactiplantibacillus plantarum* P26	*bors*	antifungal activity, surfactant production	[36]

^1^ Data not shown.

**Table 2:** Primers used for detection of virulence/functional genes.

General Function	Gene	Predicted Function	Nucleotide Sequence	Melting Temperature (°C)	Expected Amplicon Size (bp)	References
Survival at low pH	*LBA1272*	Cyclopropane FA synthase	f: GGCCGGTGTTCCACTAGTCC r: ACGTTGGGTCGATTTGACGA	60	203 pb	[38]
	*dltD*	D-alanine transfer protein	f: TTCGCCTGTTCAAGCCACAT r: ACGTGCCCTTCTTTGGTTCC		283 pb	
Folate synthesis	*folP*	Dihydropteroate synthase/dihydropteroate pyrophosphorylase	f: CCASGRCSGCTTGCATGAC r: TKACGCCGGACTCCTTTTWY	61	261 pb	
	*folK*	2-amino-4-hydroxy-6-hydroxymethyldihydropteridine diphosphokinase	f: CCATTTCCAGGTGGGGAATC r: GGGGTGGTCCAAGCAAACTT		214 pb	
Starch metabolism	*agl*	α -Glucosidase	f: GCSAAAATGCTAGCGACYMT r: CCACTGCATYGGYGTACGY	62	236 pb	
	*α-amy*	α-amilase	f: AGATCAGGCGCAAGTTCAGT r: TTTTATGGGCACACCACTCA		220 pb	
	*malL*	Oligo-1,6-glucosidase	f: TTGCCTAACAACTGGGGTTC r: ATCAACGCCTTTGTTCAACC		177 pb	
Riboflavin synthesis	*ribA*	3,4-dihydroxy-2-butanone 4-phosphate synthase/GTP cyclohydrolase II	f: TTTACGGGCGATGTTTTAGG r: CGACCCTCTTGCCGTAAATA	62	121 pb	
Virulence	*ace*	adhesion collagen protein	f: AAAGTAGAATTAGATCACAC r: TCTATCACATTCGGTTGCG	45	320 pb	[37]
	*agg*	aggregation	f: AAGAAAAAGTAGACCAAC r: AACGGCAAGACAAGTAAATA	44	1553 pb	
	*asa*	aggregation	f: GATACAAAGCCAATGTGGTTCCT r: TAAAGAGTCGCCACGTTTCACA	56	101 pb	

**Table 3:** Combination of substrates used for fermentation by selected LAB strains.

Substrate Code	Substrate Composition ^1^
1	5% WB + 5% BR
2	5% WB + 5% C
3	5% WB + 10% BR
4	5% WB + 10% C
5	10% WB + 5% BR
6	10% WB + 5% C
7	10% WB + 10% BR
8	10% WB + 10% C
9	5% WB + 5% BR + 5% C
10	10% WB + 5% BR + 10% C

^1^ WB = wheat bran, BR = red beetroot, C = carrots.

**Table 4:** Functional genes present in the genome of tested bacteria.

Strain	Gene							
	*LBA1272*	*dltD*	*folP*	*folK*	*agl*	*α-amy*	*malL*	*ribA*
*Lb. plantarum* P26	+	−	+	−	+	+	−	+
*Lb. plantarum* P35	+	−	+	−	+	+	−	+
*Lb. plantarum* BR9	+	−	+	−	+	+	+	+
*Lb. plantarum* CR1	+	−	+	−	+	+	+	+
*Leuc. citreum* 52	+	−	+	−	+	+	+	+
*Lb. acidophilus* IBB801	−	−	−	−	+	−	−	−
*Leuc. mesenteroides* 21.2	+	−	+	−	+	+	−	−

**Table 5:** Fermentation parameters, antibacterial activity and nutritional properties of the beverages obtained with selected strains.

Inoculum	Substrate	Sampling Time (h)	pH	Lactic Acid (mg/mL)	log CFU/ml		Total Free Phenolics	Flavonoids (µg RE/mL)	DPPH Scavenging Effect (%)	Inhibitory Activity (AU/mL)
					MRS	VRBG	(µg GAE/mL)			
*L. plantarum* BR9	5% WB + 10% BR	0	6.3 ± 0.1	nd	6.0 ± 0	nd	121 ± 10	110 ± 22	33 ± 2	nd
		3	6.0 ± 0.1	nd	6.8 ± 0.2	nd	132 ± 2	110 ± 7	40 ± 3	nd
		6	5.0 ± 0.3	1.2 ± 0.3	7.8 ± 0.2	1.2 ± 0.6	148 ± 8	109 ± 12	50 ± 2	nd
		9	3.8 ± 0.1	4.5 ± 0.1	8.3 ± 0.2	nd	158 ± 4	102 ± 9	51 ± 4	nd
		12	3.5 ± 0.1	7.0 ± 0.3	8.7 ± 0.3	nd	161 ± 1	101 ± 6	50 ± 1	nd
		24	3.3 ± 0	11.0 ± 0.2	9.1 ± 0.2	nd	180 ± 6	120 ± 1	54 ± 2	nd
		1week	-	-	8.6 ± 0.3	nd	-	-	-	-
	5% WB + 10% C	0	6.3 ± 0.2	nd	5.9 ± 0.1	0.6 ± 0.1	81 ± 1	44 ± 2	35 ± 5	nd
		3	6.0 ± 0	nd	6.6 ± 0.1	1.3 ± 0.1	92 ± 3	52 ± 6	29 ± 3	nd
		6	5.1 ± 0.3	nd	7.4 ± 0.4	1.8 ± 0.2	100 ± 3	49 ± 5	27 ± 4	nd
		9	3.8 ± 0.2	4.4 ± 0.2	8.4 ± 0.3	0.8 ± 0.1	114 ± 2	47 ± 1	22 ± 2	nd
		12	3.6 ± 0.1	6.3 ± 0.1	8.8 ± 0.4	nd	119 ± 0	59 ± 5	23 ± 2	nd
		24	3.3 ± 0	11.1 ± 0.1	8.9 ± 0.4	nd	149 ± 2	69 ± 7	28 ± 3	nd
		1 week	-	-	7.5 ± 0.2	nd	-	-	-	-
*L. plantarum* P35	5% WB + 10% BR	0	6.1 ± 0	nd	7.4 ± 0.2	nd	128 ± 7	116 ± 8	41 ± 1	nd
		3	4.9 ± 0.1	nd	7.7 ± 0.1	nd	148 ± 10	123 ± 8	50 ± 5	100
		6	3.9 ± 0	0.8 ± 0.1	8.4 ± 0.5	nd	152 ± 10	110 ± 9	48 ± 3	100
		9	3.5 ± 0.1	3.8 ± 0.4	8.6 ± 0.3	nd	155 ± 10	117 ± 2	48 ± 6	100
		12	3.4 ± 0	5.5 ± 0.5	8.8 ± 0.1	nd	156 ± 9	114 ± 3	49 ± 2	100
		24	3.2 ± 0.1	12.1 ± 0.1	8.7 ± 0.2	nd	188 ± 8	123 ± 3	51 ± 1	100
		1 week	-	-	8.8 ± 0.1	nd	-	-	-	-
	5% WB + 10% C	0	6.0 ± 0	nd	8.2 ± 0.2	nd	84 ± 6	35 ± 1	20 ± 4	nd
		3	4.7 ± 0.1	nd	8.6 ± 0.1	nd	95 ± 1	36 ± 1	18 ± 1	nd
		6	3.8 ± 0	3.7 ± 0.5	8.9 ± 0.5	nd	107 ± 2	46 ± 2	19 ± 3	100
		9	3.5 ± 0.1	6.7 ± 0.5	9.6 ± 0.9	nd	114 ± 3	53 ± 7	23 ± 2	100
		12	3.3 ± 0.1	8.8 ± 0.5	9.8 ± 1.0	nd	123 ± 5	58 ± 7	22 ± 3	100
		24	3.2 ± 0	15.0 ± 1.8	9.8 ± 1.1	nd	147 ± 3	75 ± 1	25 ± 1	100
		1 week	-	-	8.9 ± 0.4	nd	-	-	-	-
*L. acidophilus* IBB801	5% WB + 10% BR	0	6.2 ± 0.1	nd	6.9 ± 0.3	nd	112 ± 2	83 ± 3	33 ± 3	nd
		3	5.6 ± 0.1	nd	7.0 ± 0.1	nd	131 ± 1	92 ± 4	39 ± 6	nd
		6	4.9 ± 0	1.5 ± 0.2	7.7 ± 0.1	2.4 ± 0.2	138 ± 4	83 ± 1	40 ± 2	nd
		9	4.1 ± 0.1	3.0 ± 0.4	7.9 ± 0.2	2.5 ± 0.3	146 ± 5	95 ± 5	43 ± 4	nd
		12	3.9 ± 0.1	4.2 ± 0.2	8.0 ± 0.1	nd	160 ± 7	103 ± 2	42 ± 1	100
		24	3.6 ± 0.1	7.4 ± 1.1	8.1 ± 0.2	nd	173 ± 1	136 ± 6	44 ± 3	100
		1 week	-	-	8.2 ± 0.3	nd	-	-	-	-
	5% WB + 10% C	0	6.1 ± 0	nd	6.9 ± 0.1	nd	68 ± 1	40 ± 3	32 ± 3	nd
		3	5.5 ± 0.1	nd	7.0 ± 0.2	nd	88 ± 3	41 ± 1	25 ± 1	nd
		6	4.7 ± 0.2	1.9 ± 0.1	7.6 ± 0.1	2.0 ± 0.1	92 ± 4	39 ± 2	18 ± 2	nd
		9	4.1 ± 0.1	3.3 ± 0.3	7.9 ± 0.3	2.3 ± 0.2	105 ± 6	47 ± 1	18 ± 3	100
		12	3.8 ± 0	4.2 ± 0.4	8.0 ± 0.1	2.8 ± 0.2	123 ± 2	44 ± 3	20 ± 1	200
		24	3.4 ± 0	8.0 ± 0.9	8.1 ± 0.1	nd	150 ± 2	60 ± 2	33 ± 1	100
		1 week	-	-	8.4 ± 0.4	nd	-	-	-	-
*L. plantarum* BR9 + *L. acidophilus* IBB801	5% WB + 10% BR	0	6.2 ± 0.1	nd	7.0 ± 0.1	nd	114 ± 4	95 ± 2	46 ± 5	nd
		3	5.9 ± 0	nd	7.0 ± 0.1	nd	128 ± 3	111 ± 1	52 ± 2	nd
		6	5.2 ± 0.2	1.5 ± 0.2	7.6 ± 0.3	1.3 ± 0.1	134 ± 4	96 ± 4	51 ± 2	nd
		9	4.3 ± 0.1	3.3 ± 0.4	7.9 ± 0.3	nd	141 ± 1	122 ± 2	54 ± 6	nd
		12	3.9 ± 0.1	5.0 ± 0.5	8.2 ± 0.2	nd	148 ± 6	98 ± 5	53 ± 2	100
		24	3.4 ± 0	11.2 ± 1.2	8.3 ± 0.1	nd	165 ± 2	129 ± 4	58 ± 4	100
		1 week	-	-	8.3 ± 0.2	nd	-	-	-	-
	5% WB + 10% C	0	6.1 ± 0	nd	6.7 ± 0.2	nd	81 ± 1	53 ± 2	34 ± 5	nd
		3	5.6 ± 0.1	nd	6.8 ± 0.1	nd	89 ± 2	49 ± 2	32 ± 2	nd
		6	4.8 ± 0	1.9 ± 0.1	7.5 ± 0.3	nd	96 ± 6	40 ± 1	26 ± 3	100
		9	3.8 ± 0.2	4.8 ± 0.5	8.0 ± 0.1	nd	115 ± 5	62 ± 5	32 ± 4	200
		12	3.6 ± 0.1	6.8 ± 0.8	8.2 ± 0.1	nd	117 ± 3	58 ± 4	33 ± 3	200
		24	3.2 ± 0.2	11.5 ± 1.5	8.6 ± 0.4	nd	144 ± 4	77 ± 3	32 ± 2	100
		1 week	-	-	8.2 ± 0.5	nd	-	-	-	-
*L. plantarum* P35 + *L. acidophilus* IBB801	5% WB + 10% BR	0	6.1 ± 0.2	nd	7.3 ± 0.3	nd	119 ± 2	92 ± 2	53 ± 5	nd
		3	5.7 ± 0.1	nd	7.3 ± 0.2	nd	130 ± 4	94 ± 3	49 ± 5	nd
		6	4.8 ± 0	1.8 ± 0.1	7.8 ± 0.2	nd	135 ± 5	100 ± 8	53 ± 2	nd
		9	3.8 ± 0	4.5 ± 0.4	8.4 ± 0.1	nd	135 ± 3	126 ± 6	49 ± 6	100
		12	3.6 ± 0.1	7.2 ± 0.5	8.6 ± 0.1	nd	137± 3	105 ± 4	51 ± 2	100
		24	3.2 ± 0.1	12.3 ± 0.7	8.7 ± 0.3	nd	158 ± 2	115 ± 5	48 ± 4	100
		1 week	-	-	8.9 ± 0.6	nd	-	-	-	-
	5% WB + 10% C	0	6.1 ± 0.1	nd	7.3 ± 0.1	nd	83 ± 3	42 ± 3	32 ± 2	nd
		3	5.1 ± 0.2	nd	7.4 ± 0.1	nd	91 ± 1	56 ± 5	27 ± 3	100
		6	3.9 ± 0.1	3.6 ± 0.3	8.4 ± 0.4	nd	103 ± 5	46 ± 4	29 ± 3	200
		9	3.5 ± 0.1	7.3 ± 0.5	8.7 ± 0.1	nd	108 ± 8	60 ± 8	31 ± 5	200
		12	3.4 ± 0.1	9.5 ± 0.2	8.6 ± 0.2	nd	117 ± 2	65 ± 5	33 ± 3	100
		24	3.1 ± 0.1	13.6 ± 0.8	8.8 ± 0.2	nd	145 ± 3	104 ± 2	33 ± 4	100
		1 week	-	-	8.9 ± 0.3	nd	-	-	-	-

nd = not detected.

## Data Availability

The data presented in this study are available in the article and in the supplementary files linked to it.

## Supplementary Materials

The following supporting information can be downloaded at: https://www.mdpi.com/article/10.3390/microorganisms10122314/s1, Figure S1: PCR amplification of genomic DNA extracted from various LAB strains, using specific primers for *agg* (a), *ace* (b), and *asa* (c) virulence genes.
