# Supplementary material for: Non-Dairy Fermented Beverages Produced with Functional Lactic Acid Bacteria
Source: Microorganisms. 2022 Nov 23;10(12):2314. doi: 10.3390/microorganisms10122314 (PMC9781336; doi:10.3390/microorganisms10122314)
Supplement: Supplementary file 1 [file microorganisms-10-02314-s001.zip › microorganisms-1989580-supplementary.pdf]

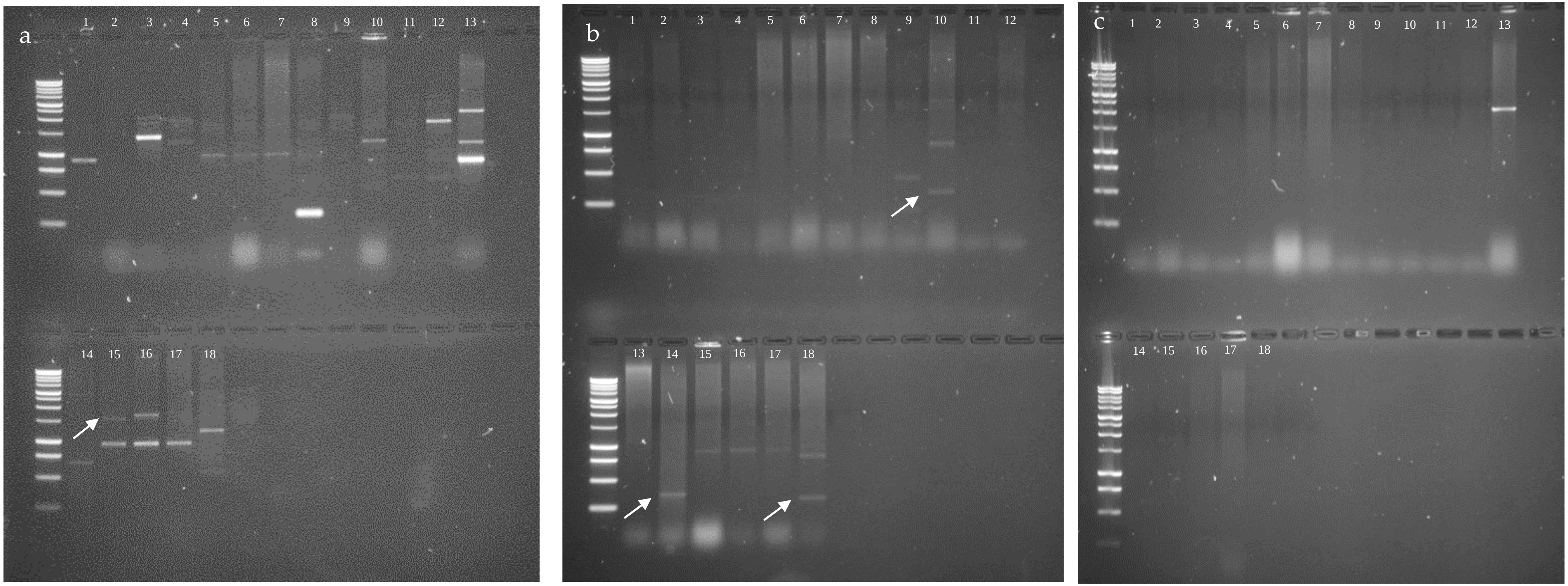

Figure S1. PCR amplification of genomic DNA extracted from various LAB strains, using specific primers for *agg* (a), *ace* (b), and *asa* (c) virulence genes. Strains used in this study are: 2 - *Lb. acidophilus* IBB801; 5 - *Lb. plantarum* BR9; 6 - *Lb. plantarum* CR1; 7 - *Lb. plantarum* P26; 9 - *Leuc. mesenteroides* 21.2; 11 - *Leuc citreum* 52; 16 - *Lb. plantarum* P35. Arrows indicate the specific amplicons, found in other LAB strains .
